# Power to the protein: enhancing and combining activities using the Spy toolbox

**DOI:** 10.1039/d0sc01878c

**Published:** 2020-07-03

**Authors:** Anthony H. Keeble, Mark Howarth

**Affiliations:** a Department of Biochemistry , University of Oxford , South Parks Road , Oxford , OX1 3QU , UK . Email: mark.howarth@bioch.ox.ac.uk ; Tel: +44 (0)1865 613200

## Abstract

Proteins span an extraordinary range of shapes, sizes and functionalities. Therefore generic approaches are needed to overcome this diversity and stream-line protein analysis or application. Here we review SpyTag technology, now used in hundreds of publications or patents, and its potential for detecting and controlling protein behaviour. SpyTag forms a spontaneous and irreversible isopeptide bond upon binding its protein partner SpyCatcher, where both parts are genetically-encoded. New variants of this pair allow reaction at a rate approaching the diffusion limit, while reversible versions allow purification of SpyTagged proteins or tuned dynamic interaction inside cells. Anchoring of SpyTag-linked proteins has been established to diverse nanoparticles or surfaces, including gold, graphene and the air/water interface. SpyTag/SpyCatcher is mechanically stable, so is widely used for investigating protein folding and force sensitivity. A toolbox of scaffolds allows SpyTag-fusions to be assembled into defined multimers, from dimers to 180-mers, or unlimited 1D, 2D or 3D networks. Icosahedral multimers are being evaluated for vaccination against malaria, HIV and cancer. For enzymes, Spy technology has increased resilience, promoted substrate channelling, and assembled hydrogels for continuous flow biocatalysis. Combinatorial increase in functionality has been achieved through modular derivatisation of antibodies, light-emitting diodes or viral vectors. In living cells, SpyTag allowed imaging of protein trafficking, retargeting of CAR-T cell killing, investigation of heart contraction, and control of nucleosome position. The simple genetic encoding and rapid irreversible reaction provide diverse opportunities to enhance protein function. We describe limitations as well as future directions.

## Abstract 2

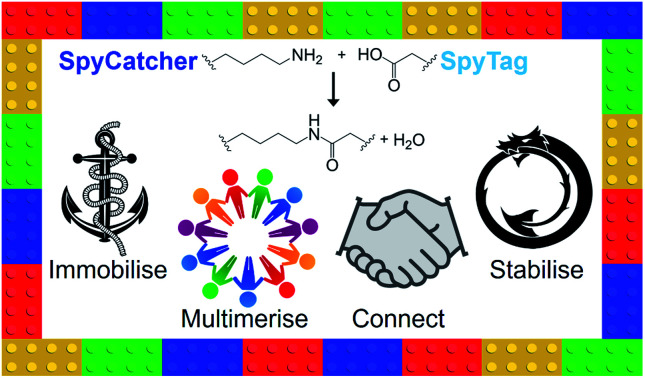
A peptide with simple and selective reactivity expands the function of proteins, from single molecule analysis to potential clinical application.

## 1. Fundamentals of SpyTag/SpyCatcher technology

### 1.1 Spontaneous amidation: kinetic and thermodynamic features

**Fig. 1:**
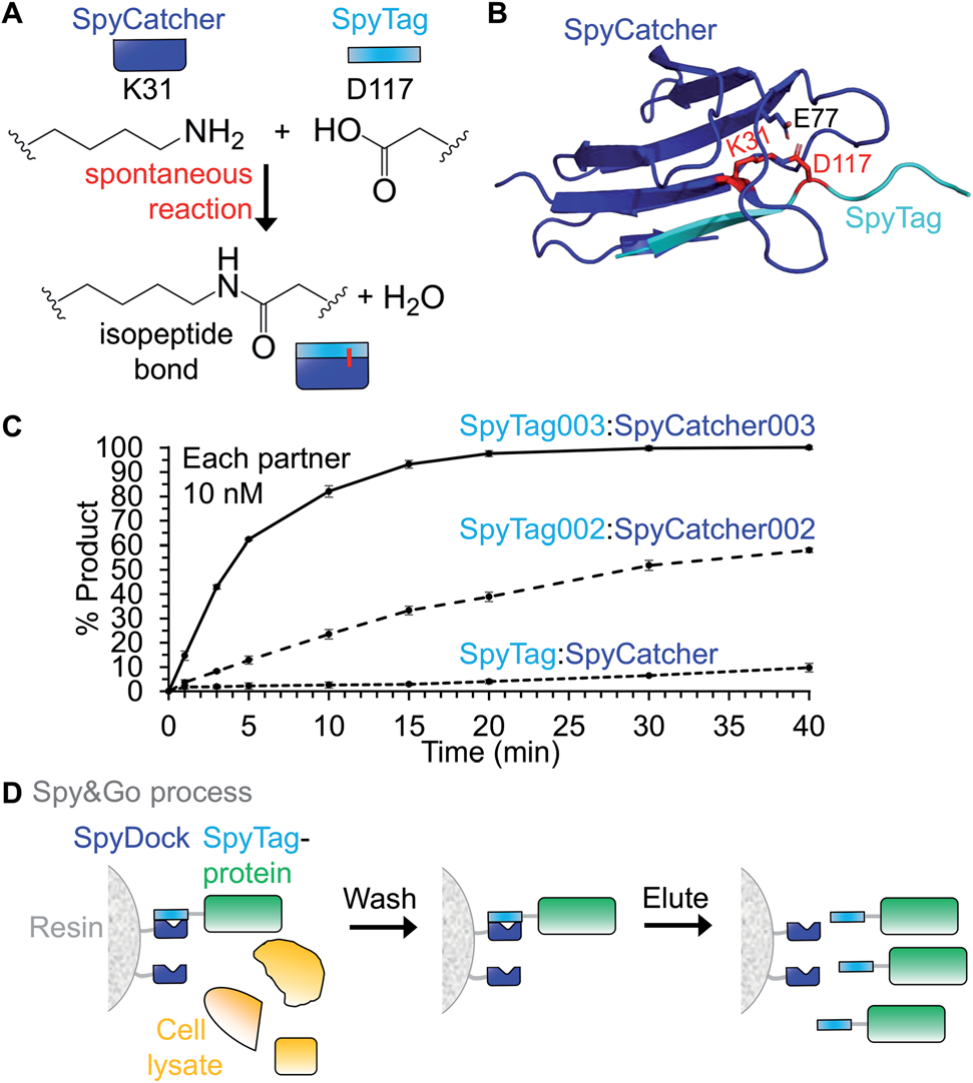
Fundamentals of the SpyTag system. (A) Spontaneous isopeptide bond formation by reaction of Lys31 of SpyCatcher with Asp117 of SpyTag (numbering from PDB 2X5P). (B) Structural basis of reaction of SpyTag (cyan) with SpyCatcher (dark blue). Reactive residues are marked in red in stick format. E77, also in stick format, transfers protons to facilitate reaction (based on PDB ; 2X5P and ; 4MLI). (C) Reaction rate of different Spy generations at low concentration. 10 nM SpyCatcher variants were incubated for the indicated time with 10 nM SpyTag-fusion protein variants at pH 7.0, 25 °C (mean ± 1 s.d., *n* = 3, adapted from ref. 6). (D) Spy&Go purification. SpyDock on resin is incubated with cell lysate containing a fusion to a SpyTag variant. Non-specific proteins can be washed away and the Spy-fusion eluted using imidazole.

SpyTag/SpyCatcher is a protein coupling approach created by splitting the CnaB2 domain from the fibronectin binding protein FbaB from *Streptococcus pyogenes*.^1^ CnaB2 spontaneously forms an intramolecular isopeptide bond between Lys31 and Asp117 (Fig. 1A). SpyCatcher is a 113-residue protein and contains the reactive Lys31. The second part, dubbed SpyTag, is a 13-residue peptide that contains the reactive Asp117 (Fig. 1A). Upon mixing, SpyTag and SpyCatcher associate and spontaneously carry out an amidation reaction promoted by the SpyCatcher residue Glu77, to form an intermolecular isopeptide bond (Fig. 1A and B). Spontaneous amidation between SpyTag/SpyCatcher occurs in a wide-range of temperatures (4–37 °C), buffers and pH values.^1^ SpyTag and SpyCatcher can be genetically fused to the N- or C-terminus of proteins, and in some cases within internal loops of proteins.^2^ Neither moiety contains any cysteine and so it is simple to use in different cellular locations. The reaction is irreversible and proceeds to >99% conversion.^1^,^3^ This approach allows specific covalent coupling of proteins both *in vitro* and in cells from various species.^2^

### 1.2 Infinite affinity: concept, engineering and potential

Assembling macromolecular complexes using non-covalent interactions has limited kinetic stability due to the finite value of their dissociation rate-constants. Some covalent interactions, such as disulfide bonds, can rapidly rearrange. However, as far as we have been able to measure, the isopeptide bond between SpyTag and SpyCatcher is irreversible.^1^ However, such stable reaction has limited utility if the reaction occurs slowly and only in the presence of high concentrations of each partner. Therefore, it is essential to consider each rate-constant. SpyTag and SpyCatcher form an initial non-covalent complex (with association rate-constant *k*_on_ and dissociation rate-constant *k*_off_), before reacting with a rate-constant *k*_2_. The best that could happen is a situation termed infinite affinity, where *k*_on_ is diffusion-limited and *k*_2_ is much greater than *k*_off_.

The diffusion limit for association of a typical protein:protein complex is 10^5^–10^6^ M^–1^ s^–1^.^4^ Thus, an infinite affinity binding reagent should associate with a *k*_on_ of 10^5^–10^6^ M^–1^ s^–1^ and react with a second order rate-constant of 10^5^–10^6^ M^–1^ s^–1^. Through phage display selection and rational design, the SpyTag003/SpyCatcher003 pair now has kinetics approaching infinite affinity. SpyTag003/SpyCatcher003 react with a rate of 5.5 × 10^5^ M^–1^ s^–1^, about 400-fold faster than SpyTag/SpyCatcher (1.4 × 10^3^ M^–1^ s^–1^) (Fig. 1C).^5^,^6^ Even at low protein concentrations (10 nM), SpyTag003/SpyCatcher003 react close to completion in 15 min, conditions under which little of the original SpyTag/SpyCatcher reacts (Fig. 1C).^6^ This rapid reaction opens new potential applications, including for intracellular coupling of poorly expressing proteins on biologically relevant time-scales and enhanced western blot detection.^6^

### 1.3 Spy&Go: affinity purification by non-reactive SpyCatcher

An ideal purification tag enables simple and efficient isolation of a protein construct from complex mixtures, but should also enhance the downstream function of that construct. To avoid limitations of the His_6_-tag, such as the toxicity of Ni^2+^, we established the use of SpyTag for protein purification. Spy&Go employs a non-reactive SpyCatcher mutant (SpyDock) to enable purification of proteins containing reactive SpyTags (original, SpyTag002 or SpyTag003) (Fig. 1D).^7^ Cell lysate or supernatant containing the SpyTag-linked protein of interest is mixed with SpyDock resin, impurities washed away, and the SpyTag-fusion eluted using high concentrations of imidazole. Spy&Go was shown for purification of proteins from bacterial or mammalian expression. Purification of a dual tagged (His_6_-tag/SpyTag) maltose binding protein by Spy&Go gave a higher purity (98.9 ± 0.5%) than *via* Ni-NTA purification (66.4 ± 1.9%).^7^ Proteins with either N- or C-terminally fused SpyTag as well as with SpyTag inserted in an internal loop could be purified from lysates, with binding capacities of 4–13 mg protein per mL of resin. SpyDock resin was able to be regenerated multiple times and could be stored in 20% ethanol.^7^ One of Spy&Go's key initial applications has been the purification of malaria antigens, which can then been be coupled to VLPs as vaccine candidates without an anti-His-tag immune response.^7^

## 2. Application areas

### 2.1 Anchoring to surfaces or particles

Classical approaches for anchoring proteins to a surface are hydrophobic adsorption or reaction with one of the many surface amines (from the N-terminus or Lys side-chains).^8^ Such approaches lack precision in orientation and often impair protein function.^9^ Alternatively, proteins with a natural or artificially introduced surface Cys can be coupled to maleimide or iodoacetyl groups.^8^ However, thiol-mediated coupling faces challenges from: (i) competition between coupling and disulfide bond formation, (ii) free Cys interfering with protein secretion, or (iii) promoting misfolding in proteins already containing disulfide bonds.^9^

**Fig. 2:**
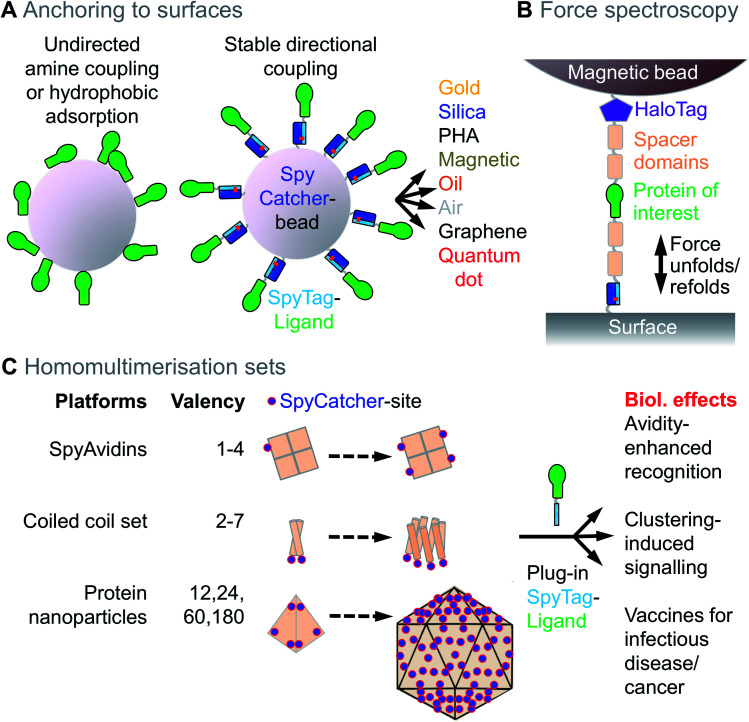
Anchoring and homomultimerisation with Spy technology. (A) Schematic of unoriented anchoring of a protein to a bead (left) *versus* oriented SpyTag/SpyCatcher-mediated anchoring (right). Different surfaces bridged using Spy technology are shown. PHA = Polyhydroxyalkanoate. (B) Force spectroscopy using SpyTag anchoring. Sample scheme for directional tethering of a protein of interest (green) between a surface and magnetic tweezers, using SpyTag, HaloTag and spacer domains, to allow repeated folding and unfolding tests. (C) Toolbox for homomultimerisation. SpyAvidins, coiled coils, or protein nanoparticles may be genetically fused to SpyCatcher (marked as a blue dot for ease of visualisation). Addition of SpyTag-fused ligand allows testing of how different multimerisation states change biological effects.

For attachment to magnetic beads, the advantage of oriented SpyTag-mediated anchoring of single-domain antibodies was shown, when compared to non-specific attachment by chemical activation of carboxylic acids (Fig. 2A).^10^ SpyTag-mediated anchoring has been applied for protein functionalisation of a range of surfaces, including gold nanoparticles^11^ and quantum dots^12^ (Fig. 2A). Anchoring onto a single layer of graphene using SpyTag has been developed for enhancing cryo-electron microscopy structure determination at atomic resolution.^13^ SpyTag-linked proteins may be anchored to silica particles assembled through biomimetic silicification^14^ or to plastic particles (polyhydroxyalkanoate, PHA) synthesised inside the cell^15^ (Fig. 2A).

To target to the air/water interface, Lynne Regan's group have shown how hydrophobins can still form a monolayer at this interface when linked to SpyTag.^16^ Hydrophobin-SpyTag can also assemble proteins of interest around oil droplets, where the particles remained monodisperse for weeks at room temperature.^17^ Air bubbles can be nucleated inside cells with genetically-encoded acoustic nanocapsules, which can be decorated using SpyTag/SpyCatcher and provide probes for targeted ultrasound.^18^

To address the imprecision of amine-mediated attachment, proteins have been genetically fused to the AviTag peptide and site-specifically biotinylated using BirA.^19^,^20^ Biotinylated proteins can then be coupled to streptavidin-linked surfaces, since streptavidin:biotin is one of the strongest non-covalent interactions. However, the streptavidin/biotin linkage is not irreversible,^21^ BirA reaction may not reach completion, and biotinylating a protein adds two steps to any pipeline: (i) performing the biotinylation reaction, and (ii) removing free biotin to avoid competition for streptavidin binding sites. Reversibility of streptavidin:biotin is an acute problem when force is applied to the interaction (*e.g.* ∼60 pN for > 1 minute when studying protein unfolding),^22^ at elevated temperatures, or for long-term storage. Researchers studying the force-dependence of protein interactions and protein folding have sought an interaction that was resistant to force and inextensible. The isopeptide bond runs between the Lys near the N-terminus of SpyCatcher to the Asp in the C-terminus of SpyTag, such that the force passes through the isopeptide bond and does not unfold the rest of the SpyCatcher domain.^23^,^24^ Therefore, SpyTag/SpyCatcher has become a common tool for force spectroscopy,^1^ complementing HaloTag's covalent interaction with alkyl halide ligands^22^ (Fig. 2B). Proteins for stretching can be fused to SpyTag at any accessible site and SpyCatcher is typically anchored to the solid-phase through an N-terminal Cys (Fig. 2B). Such SpyTag-anchored stretching has been applied, for example, for testing the mechanical basis of human hearing^25^ or for studying the folding pathway of computationally-designed membrane proteins.^26^ Force may be measured after SpyTag anchoring using atomic force microscopy (AFM), optical tweezers, or magnetic tweezers.^27^

### 2.2 Control of protein multimerisation state

Natural proteins take on a huge range of multimerisation architectures,^28^ from the familiar dimers and tetramers, up to icosahedral architectures with 60 or 180 copies. This multimerisation can have major effects on protein behaviour. Ligand dimerisation is a common way to activate cell signalling.^29^ Ligand tetramerisation provides avidity, revealing natural low affinity interactions, *e.g.* MHC multimers identify antigen-specific T cell populations in infection or cancer.^30^ Changing the multimerisation state of a protein of interest has often depended upon painstaking genetic fusion or tetramerisation of a biotinylated variant using streptavidin.^31^ Modular covalent assembly brings the potential to generate one protein of interest bearing SpyTag and then immediately access a toolbox of other protein scaffolds with defined architectures (Fig. 2C).

Low valency assemblies with dihedral symmetry can be accessed through “SpyAvidins” (Fig. 2C). We showed that streptavidin subunits could be fused with SpyCatcher and chimaeric tetramers generated with precisely 1, 2, 3 or 4 SpyCatcher copies.^31^ Thereby, ligands could be clustered, as well as interfaced with biotinylated ligands.

To access other low valencies using cyclic symmetry, we prepared a set of coiled coil architectures,^7^ harnessing structures known in nature or computationally designed by Dek Woolfson's group^32^ (Fig. 2C). We applied this coiled coil set, from dimer up to heptamer, to follow valency-dependence of Death Receptor 5 activation of apoptosis.^7^

To access higher valencies, we and others have used icosahedral protein architectures (Fig. 2C). SpyCatcher fused to Dodecin from *Mycobacterium tuberculosis* is a stable 12-mer, which can be quantitatively coupled to SpyTag-fused ligands.^33^ SpyCatcher-linked Ferritin has 24 subunits and was used for multimerising tumour neoantigens.^34^ SpyCatcher-mi3 is a modified version of computationally-designed 60-mer, which expresses efficiently in *Escherichia coli* and allowed simple coupling with blood-stage and transmission-blocking malaria antigens.^35^ SpyCatcher-AP205, based on a phage capsid, has 180 subunits and can be coupled to antigens related to HIV, tuberculosis and cancer.^36^,^37^ Clustering on viral-like particles leads to a major increase in immunogenicity and may enhance vaccine development for a range of diseases^9^ (Fig. 2C). Such SpyTag-based clustering has also been applied to bacterial microcompartments^38^ or live viruses, *e.g.* oncolytic Herpes Simplex Virus,^39^ the filamentous viruses Potato Virus X^40^ and Tobacco Mosaic Virus,^41^ lentivirus^42^ or Adeno-associated Virus (AAV).^43^,^44^ Here there is particular interest to change the viral tropism, including retargeting the virus to tumours.

For multimers that can potentially extend infinitely, the first approach using SpyTag was 1D fibres made from the bacterial amyloid-forming protein CsgA.^45^,^46^ CsgA-SpyTag was secreted by *E. coli* to form a living material and generate nanofibres decorated with gold nanoparticles or quantum dots.^46^ The fibres form amyloid networks with extreme stability: the Joshi lab showed how CsgA-SpyCatcher could be isolated from *E. coli* as a nanoporous mat, survive washing with organic solvent and sodium dodecyl sulfate (SDS), and subsequently still react with SpyTag-fusions.^47^ 2D surfaces functionalisable *via* SpyTag (either *in vitro* or covering living cells) came from fusion to S-layer proteins.^48^ 3D structures applying SpyTag/SpyCatcher were first assembled by the groups of David Tirrell and Frances Arnold: hydrogels form upon mixing one protein bearing multiple SpyTags with another protein bearing multiple SpyCatchers.^49^ This gelation enabled stem cell encapsulation; in contrast to other chemistries for hydrogel formation, each component can be precisely functionalised (*e.g.* with integrin binding sites or matrix metalloproteinase cleavage sites) and the bio-orthogonal reaction leads to high viability of different cell-types.^49^ 3D networks can also be assembled after chemical coupling of SpyTag to polymers, for polyethylene glycol (PEG) networks joined by light-induced radical formation.^50^

### 2.3 Multiplexing protein function

Proteins with different functionality can often be connected by genetic fusion. However, challenges arise from:

– Increase in folding complexity.

– Mismatch in multimerisation state of each unit.

– Requirement for different post-translational or synthetic chemical modification of each moiety.^9^

**Fig. 3:**
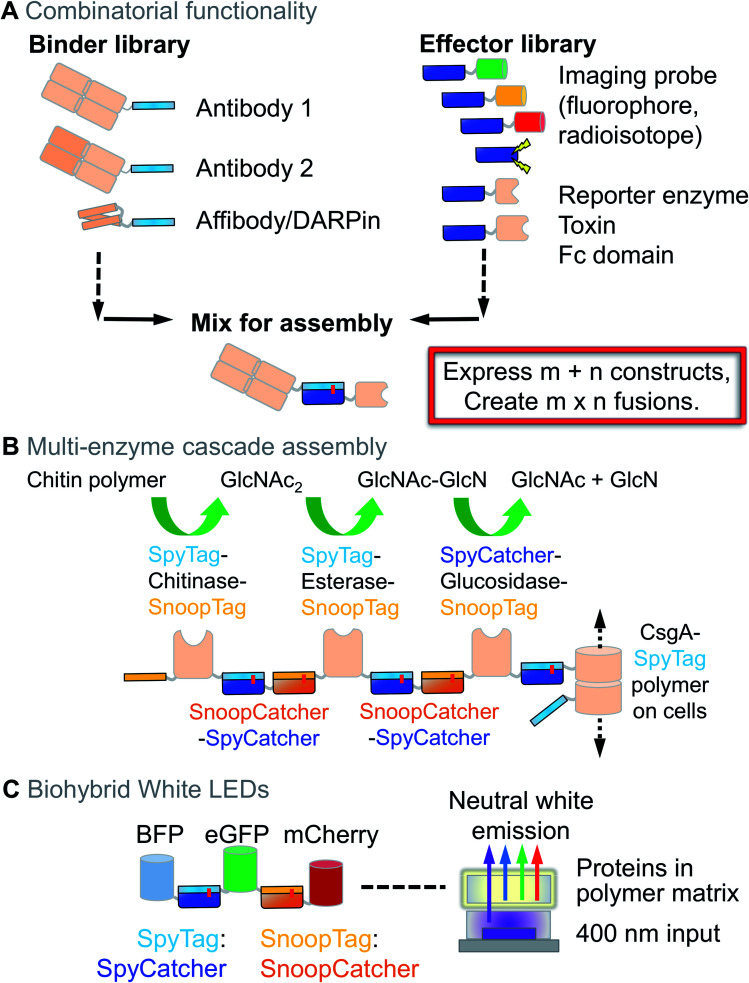
Combining functions using Spy technology. (A) Combinatorial antibody decoration. A binder library bearing SpyTag can be mixed with a library of effectors bearing SpyCatcher, leading to rapid expansion of functional properties. (B) Multi-enzyme cascade from Spy and Snoop assembly. CsgA-SpyTag forms filaments extending from the cell-surface. The filaments act as a solid-phase, allowing sequential coupling of enzymes for chitin degradation using orthogonal reaction of SpyTag/SpyCatcher and SnoopTag/SnoopCatcher. GlcNAc = *N*-acetylglucosamine; GlcN = glucosamine. (C) White LED assembly. 3 different fluorescent proteins are ligated by SpyTag/SpyCatcher and SnoopTag/SnoopCatcher reaction, enabling efficient Förster resonance energy transfer (FRET). Encapsulation in a matrix and illumination at 400 nm leads to stable neutral white light emission. BFP = blue fluorescent protein; eGFP = enhanced green fluorescent protein.

Therefore there are many situations where modular coupling is preferable. This is especially urgent given the revolutions in Omics and Personalised Medicine, where people are looking for scalable approaches to be applied on 1000 to 100 000 targets. For example, the Human Proteome Project has long had a goal of specific binding reagents for every protein in the human proteome.^51^ For each of ∼20 000 reagents, one would desire a version linked to *e.g.* 8 different fluorophores as well as a probe for ELISA and *in vivo* imaging (Fig. 3A). With genetic fusion, one requires 20 000 × 10 constructs to be cloned, expressed and purified, which is impractical even for the largest company. With modular coupling, one requires 20 000 + 10 constructs (Fig. 3A). This concept was put into practice with a cell-free *in vitro* transcription and translation mix, where binding reagents linked to SpyTag were multiplexed with SpyCatcher-linked fluorescent proteins, reporter enzymes or toxins (Fig. 3A).^52^ Ron Geyer's lab has developed this concept for multiplexing of antibody formats.^53^ Modular assembly with SpyTag/SpyCatcher has also been used to make heterodimeric binders ligating either different cell-surface receptors^54^ or different regions of the same receptor.^55^

Multicomponent assembly can be enhanced through combining SpyTag/SpyCatcher with the orthogonal SnoopTag/SnoopCatcher pair, as we applied for 9 ligation steps on a Sepharose solid-phase. The resultant affibody/nanobody teams were tested for synergy in cancer cell killing.^3^ CsgA amyloid can also be used as a solid-phase, for sequential assembly of a multi-enzyme pathway for chitin degradation (Fig. 3B).^56^ Our group extended 3D network assembly by chemical coupling of SpyTag to the polysaccharide hyaluronic acid, allowing gelation by a protein containing two SpyCatchers.^57^ Using SnoopTag, these hydrogels were independently functionalised with adhesion proteins to modulate behaviour of tumour spheroids.^57^ Combining Spy and Snoop pairs also allowed layer-by-layer nanoassembly to harvest uranium from seawater.^58^,^59^

To address the environmental impact of conventional light-emitting diode (LED) phosphors, a white LED was assembled through ligation of fluorescent proteins emitting in different parts of the spectrum (Fig. 3C).^60^ Similarly, to increase solar energy conversion, plant light harvesting complexes have recently been covalently combined with reaction centres from a purple photosynthetic bacterium to give complementary light absorption.^61^

### 2.4 Enzyme resilience and assembly

**Fig. 4:**
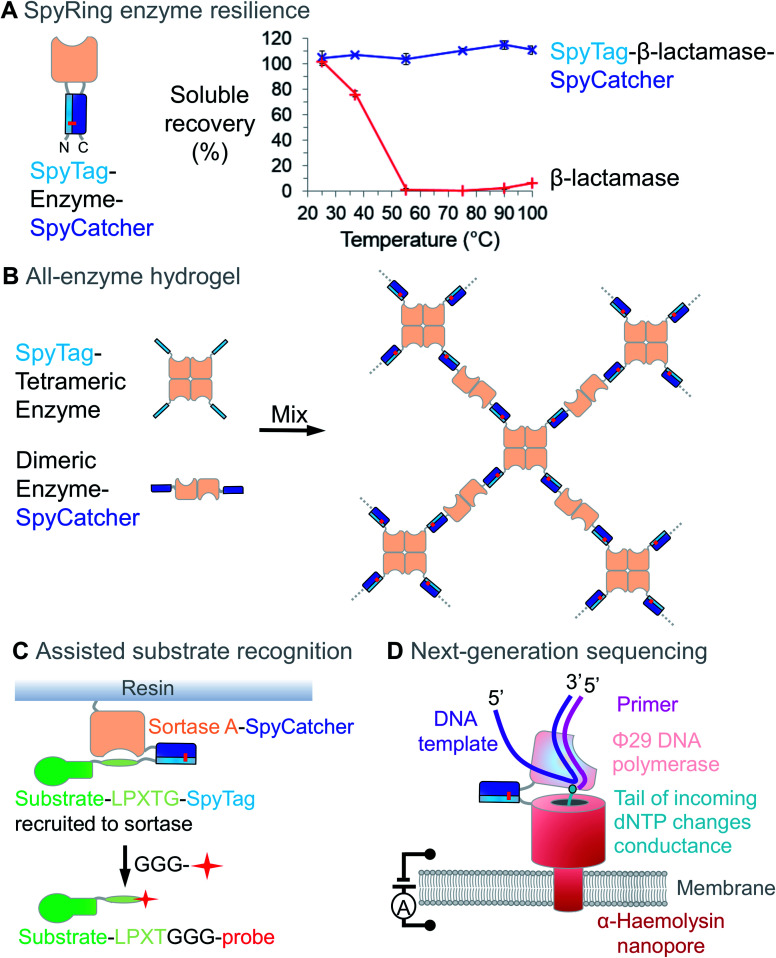
Enzyme resilience and connection using Spy technology. (A) Cyclisation with SpyTag/SpyCatcher can increase enzyme resilience. SpyTag-β-lactamase-SpyCatcher forms an intramolecular isopeptide bond. Upon heating at the indicated temperature for 10 min, aggregated protein was removed by centrifugation and soluble fraction determined (mean ± 1 s.d., *n* = 3; adapted from ref. 63). (B) All-enzyme hydrogel. Mixing SpyTag-tetrameric enzyme with SpyCatcher-dimeric enzyme leads to rapid gelation and stable catalytic function. (C) Assisted substrate recognition. Sortase-SpyCatcher efficiently recruits SpyTag-linked substrate, with release triggered by an oligoglycine-linked biophysical probe. (D) Next-generation sequencing. SpyTag can anchor enzymes to nanopores, with single-molecule detection of DNA sequence from the effect of the tail-modified deoxynucleoside triphosphate (dNTP) on the current.

Enzymes often require a dynamic structure to bind and release their substrates, as well as for responding to regulatory inputs. Therefore, enzyme stability can be limiting in many situations. For example, enzymes used to enhance digestibility of animal feed must survive treatment with high-pressure steam.^62^ Since termini are often the most flexible part of a protein, we tested how locking the termini together through SpyTag/SpyCatcher (to give SpyRings, Fig. 4A) could change the resilience of an enzyme. We were surprised to find that the effect was often dramatic, with an enzyme such as β-lactamase retaining nearly all of its solubility and catalytic activity following boiling, if cyclised in this way (Fig. 4A).^63^ Similar effects on resilience were found for SpyRing versions of phytase,^64^ xylanase^65^ and luciferase.^66^ Apart from temperature, increased tolerance to organic solvents and denaturant has been found upon SpyRing cyclisation.^67^

Another approach to enhance stability and performance has been to encapsulate enzymes in protein cages. Pamela Silver's lab fused two enzymes in indigo biosynthesis *via* SpyTag/SpyCatcher on the inside of MS2 phage nanoparticles. This encapsulation enhanced indigo production inside cells, as well as increasing enzyme stability after 1 week from 95% compared to 5% for free enzymes.^68^ Wen-Bin Zhang's lab used p53's dimerisation domain and SpyTag/SpyCatcher to create protein catenanes, generating a dihydrofolate reductase with increased thermal and proteolytic stability.^69^

Hydrogels have also been assembled using SpyTag/SpyCatcher with the enzymes themselves as the construction material. These enzyme networks showed efficient catalytic conversion and good stability for continuous flow biocatalysis (Fig. 4B).^70^,^71^ Bringing together 3 enzymes into a hydrogel with SpyTag/SpyCatcher linked to elastin-like polypeptides increased the yield in Vitamin K2 biosynthesis.^72^

To improve enzyme performance, SpyTag/SpyCatcher may facilitate substrate recruitment. Sortase recognises an –LPXTG motif on a substrate protein and directs ligation to oligoglycine-bearing probes (Fig. 4C). Sortase is often used at concentrations similar to that of its substrates and efficiency can be limited by sortase's low affinity for –LPXTG. Using –LPXTG linked to SpyTag and Sortase linked to SpyCatcher, the Tsourkas lab enhanced this substrate docking, improving reaction speed and yield (Fig. 4C).^73^ Precise positioning and long-term assembly are also important for next-generation DNA sequencing, where SpyTag positions DNA polymerase adjacent to a nanopore and current change reads out nucleic acid sequence (Fig. 4D).^74^,^75^

### 2.5 Cellular applications

**Fig. 5:**
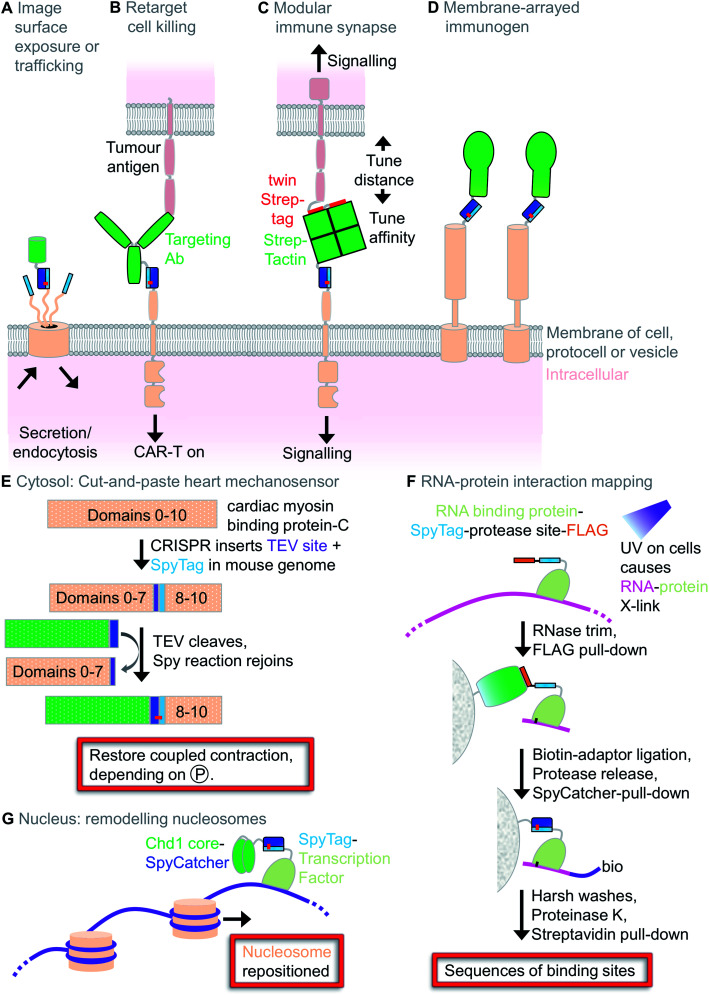
Cellular applications of Spy technology. (A) Imaging surface exposure or protein trafficking. SpyCatcher linked to a fluorescent protein or dye detects surface protein exposure or cellular dynamics following endocytosis. (B) Retargeting cell killing. CAR-T cells expressing SpyCatcher can be directed *via* a SpyTag-linked antibody to kill cancer cells. (C) Modular immune synapse. One cell expresses twin Strep-tag, while the other cell expresses SpyTag. Cell–cell communication is tuned by soluble Strep-Tactin-SpyCatcher. (D) Membrane-arrayed immunogen. SpyTag-linked membrane proteins on the outside of cells or outer membrane vesicles can react with SpyCatcher-fused antigens to induce a strong immune response. (E) Cut-and-paste heart mechanosensor. Knock-in mice allow modification of a mechanosensor in permeabilised heart muscle. (F) RNA–protein interaction mapping. SpyCLIP procedure, harnessing the stability of SpyTag/SpyCatcher for identification of RNA-binding sites of a protein of interest. (G) Remodelling nucleosomes. Modular linkage of a transcription factor to a chromatin-remodelling factor (Chd1 core).

The first paper on SpyTag showed how the peptide could be fused to a cell-surface protein of interest (ICAM-1) for specific labelling on live cells, using SpyCatcher linked to a fluorescent dye (Fig. 5A).^1^ Subsequent major advances were imaging of channelrhodopsins inside living *Caenorhabditis elegans*^76^ and super-resolution fluorescent microscopy inside cells.^77^ For imaging inside *Saccharomyces cerevisiae*, direct fusion to GFP can be problematic for various plasma membrane proteins but labelling *via* SpyTag/SpyCatcher improved localisation.^78^ Fusing SpyTag to a voltage-sensitive dye was applied for fluorescent imaging of neuronal action potentials.^79^ Various elegant studies used SpyTag to establish membrane translocation in bacterial inner and outer membranes (Fig. 5A).^80^,^81^

A revolution in cancer treatment in recent years has been the clinical success of immunotherapy, using either checkpoint inhibitors or Chimeric Antigen Receptor (CAR)-T cells.^82^ CAR-T cells are currently generated by lentiviral transduction, enabling redirection of T cell killing to a cancer-specific target. Modular redirection of CAR-T cells using non-covalent coiled coil assembly at the cell-surface was proposed for tunable T cell activation (*e.g.* reducing activation in the case of life-threatening cytokine storm) or redirecting T cells to new targets (in the case of immune evasion).^83^ Antibody linked to SpyTag has been injected into mice to direct SpyCatcher-expressing CAR-T cells towards ovarian cancer killing (Fig. 5B). Compared to coiled coils, this approach has the potential advantages of the higher stability of the interaction and easier analysis of CAR-T cell conjugation (since coupling survives boiling in SDS).^84^

To enhance the study of cell–cell interactions in the immune system, stable transfectants were generated bearing surface-exposed SpyTag held at various distances from the plasma membrane (Fig. 5C).^85^ Target cells expressed a surface protein bearing a twin Strep-tag. Then, questions about cell–cell interactions in terms of ligand length, valency and affinity could be addressed in a modular fashion (without making a huge range of stably transfected cell clones), through titrating in a bridging molecule of SpyCatcher linked to Strep-Tactin (Fig. 5C).^85^ Membrane decoration may also be applied for vaccine assembly: Outer Membrane Vesicles (OMVs) from attenuated *Salmonella* displaying a SpyTag-linked *E. coli* autotransporter facilitate display of SpyCatcher-linked immunogens (Fig. 5D).^86^

In terms of multicellular organisms, SpyTag has been used in silkworms,^87^*C. elegans*^76^ and recently a transgenic mouse. Samantha Harris' lab generated a mouse line with the muscle mechanosensor c-MyBP-C bearing a SpyTag and a tobacco etch virus (TEV) protease cleavage site (Fig. 5E). On detergent-permeabilised muscle cells, her group was able to cut the protein with TEV protease and paste in new SpyCatcher-linked N-terminal regions of the protein.^88^ This molecular surgery restored calcium-dependent synchronisation of muscle contraction, but depended on the phosphorylation state of the fragment. We have also used Spy technology to investigate mechanosensing in the cytosol, studying talin's activity at the interface from the extracellular matrix to the cytoskeleton. The cellular function of split talin could be reconstituted by SpyTag003/SpyCatcher003 reaction.^6^ However, non-covalent variants of SpyTag003 also allowed sufficient mechanical stability for restoration of cell shape and migration speed. Generating a panel of SpyTag003 variants with decreasing affinity for SpyCatcher003, we could then identify the point at which interaction became insufficient.^6^

Spy technology has been applied for various genetic and epigenetic applications. SpyCLIP was developed to map RNA–protein interaction in cells, taking advantage of the irreversible SpyTag interaction to reduce background and improve pull-down efficiency (Fig. 5F).^89^ Inside the nucleus, SpyTag has been fused to transcription factors for programmable changing of nucleosome positioning (Fig. 5G)^90^ or for a down-sized Cas9 for CRISPR-mediated gene editing.^91^

## 3. Summary

### 3.1 Overview

The dream of synthetic biology for redirecting biological units as reliably as components in an electronic circuit board is still a work in progress.^92^ Having simple and reliable ways to bridge proteins to each other or to non-protein components is an important part of achieving that goal. Here we have seen how the community has found many ways to employ SpyTag technology for such challenges. SpyTag may now contribute at each stage in the life of a protein: purification, analysis, and application on cells or *ex vivo*.

### 3.2 Limitations of SpyTag technology

Despite the range of uses above, SpyTag/SpyCatcher has various limitations:

(i) Coupling leaves a molecular scar: the final construct contains the ∼17 kDa SpyTag/SpyCatcher. SnoopLigase forms an isopeptide bond between two peptides, giving a smaller molecular scar (the covalently linked SnoopTagJr:DogTag), but requires higher concentration of reactants.^93^ Diverse other ligation technologies are available with the advantage of a small scar (*e.g.* sortase, unnatural amino acid) or no scar (split intein), although facing their own challenges in terms of complexity or limitation to a single terminus.^94^

(ii) Reactivity is unregulated: as soon as SpyTag and SpyCatcher collide, they can react.

(iii) Fusion tolerance: >500 SpyTag/SpyCatcher fusions have been validated (listed in the SpyBank database at https://www2.bioch.ox.ac.uk/howarth/info.htm) and we have published general guidance on the design of linkers and helpful positive and negative controls.^2^ Nevertheless, it will help to gain further experience across more compartments and organisms for when fusion to SpyTag or SpyCatcher variants may affect expression yield or perturb natural protein function.

(iv) The more established HaloTag or (strept)avidin:biotin technologies currently have greater infrastructure of commercially-available reagents.

(v) SpyTag/SpyCatcher is non-human and will induce an immune response.^36^,^95^ It is preferable to use a non-human platform for vaccines (to avoid autoimmunity). However, immunogenicity will be a challenge to SpyTag's use in therapeutics.

### 3.3 Future directions

Although there have been a range of interesting cellular studies, the majority of applications of SpyTag has been *in vitro*. The moderate reaction rate of SpyTag/SpyCatcher has limited the time resolution and labelling efficiency for proteins at low concentration. The 400-fold accelerated reaction of SpyTag003/SpyCatcher003 (ref. 6) may facilitate many more applications of covalent decoration in cells and organisms.

We recently established a panel of non-covalent SpyTag/SpyCatcher complexes with affinities spanning 20 nM to >1 μM, to test the stability requirements of a force-dependent cytosolic interaction.^6^ Being able to compare how cells respond when an interaction is irreversible, slowly dynamic or rapidly dynamic may be useful in various other biological contexts. Promising vaccine applications of Spy technology have been shown in animal models,^9^ so it will be important to evaluate how these platforms perform in the clinic. With the combination of recent advances in the underlying technology and a widening scope of applications, it will be exciting to see how the Spy toolbox develops in the future.

## Conflicts of interest

M. H. and A. H. K. are authors on patent applications covering sequences for enhanced isopeptide bond formation (UK Intellectual Property Office 1706430.4 and 1903479.2). M. H. is an author on patents for isopeptide bond formation (EP2534484), Spy&Go (1819850.7), SnoopCatcher (1509782.7) and a SpyBiotech co-founder, shareholder and consultant.
